# Bistable behaviour and medium-dependent post-translational regulation of the tryptophanase operon regulatory pathway in *Echerichia coli*

**DOI:** 10.1038/s41598-019-41856-0

**Published:** 2019-04-01

**Authors:** David I. Orozco-Gómez, Juan Eduardo Sosa-Hernández, Óscar Adrián Gallardo-Navarro, Jesús Santana-Solano, Moisés Santillán

**Affiliations:** 1 Centro de Investigación y de Estudios Avanzados del IPN, Unidad Monterrey, Vía del Conocimiento 201, Parque PIIT, 66600 Apodaca, NL Mexico; 2 Tecnologico de Monterrey, School of Engineering and Sciences, Campus Monterrey, Ave. Eugenio Garza Sada 2501, 64849 Monterrey, NL Mexico

## Abstract

The present work is aimed at studying the dynamic behaviour of the tryptopnanase (*tna*) operon, which encodes the proteins necessary to uptake and metabolise tryptophan to use it as a carbon source in the absence of glucose. To this end, we designed a micro-bioreactor capable of driving a bacterial culture to a stationary state. This allowed us to explore (at the single cell level) the *tna* operon steady-state dynamics under multiple culture conditions. Our experimental results suggest that the *tna* operon is bistable for a specific range of environmental tryptophan and glucose concentrations, and evidence that both reagents play a role on the activation of the enzyme in charge of metabolising tryptophan: tryptophanase (TnaA). Based on our experimental data and the already known regulatory mechanisms, we developed a mathematical model for the *tna* operon regulatory pathway. Our modelling results reinforce the claim that the *tna* operon is bistable, and further suggest that the activity of enzyme TnaA is regulated by the environmental levels of glucose and tryptophan via a common signalling pathway. Possible biological implications of our findings are further discussed.

## Introduction

Glucose is the favourite carbon and energy source for many living organisms, including the bacterium *Escherichia coli*. The reason for this is that alternative carbon sources usually need to be preprocessed before entering the glucose metabolic pathway, and so their metabolism is more costly from a bioenergetic perspective. A consequence of this is that the activation of the mechanisms necessary to metabolise alternative carbon sources (in several organisms but in particular in *E. coli*) is subject to tight regulation, and this results in a hierarchical consumption of carbon sources when more than one are present in the culture medium^1^. Understanding at the molecular level how *E. coli* hierarchically consumes different sources of carbon and energy has been the focus of many research efforts for at least 60 years.

Interestingly, the regulatory mechanisms of the genetic systems responsible for the transport and metabolism of carbon sources alternative to glucose have similar architectures. Their expression is down-regulated by glucose and up-regulated by the corresponding alternative carbon source. This is for instance the case of the *lac* operon in *E. coli*^2^, the *GAL* regulon in *Saccharomyces cerevisiae*^3^, and the tryptophanase (*tna*) operon in *E. coli*^4^. Furthermore, a positive feedback loop can be identified in all of these regulatory pathways, making them candidates to display bistability.

The potential bistable behaviour of the *lac* operon has been studied for decades, both experimentally and theoretically, because the concomitant switch-like behaviour provides an elegant explanation for the hierarchic preference of carbon sources by *E. coli*^5–11^. For a long time it has been clear that the lactose operon presents a bistable behaviour when it is induced with gratuitous inducers (i.e. molecules analogous to lactose that can induce the *lac* operon, but cannot be metabolized by *β* galactosidase), but whether bistability appears with the natural inducer had been controversial until recently. Zander *et al*.^11^ convincingly demonstrated that the *lac* operon is not bistable when induced with lactose.

The fact that the *lac* operon is not bistable opens the question of what evolutionary advantage, if any, positive feedback regulation confers to the gene expression network. In this regard, it is interesting to investigate whether other gene networks that regulate consumption of carbon sources alternative to glucose are bistable. For instance, it has been suggested in a theoretical study^3^ that the *GAL* regulon of *S. cerevisiae* is bistable. From the previous discussion, the present work is aimed at studying the dynamic response of the *tna* operon to environmental changes with two main objectives in mind: (1) to demonstrate bistability in this system, and (2) to improve our understanding of a recently discovered post-translational regulation of the enzyme tryptophanase, which is encoded by one of this operon genes.

The manuscript is organized as follows. In the Tryptophanase Operon section we briefly review the known mechanisms of the tryptophanase-operon regulatory pathway. In the Materials and Methods section we describe the material and experimental methods employed to study the *tna* operon dynamic behaviour, and introduce a deterministic mathematical model for this operon. The experimental results are presented in the section with such name. In the section Modelling Results we analyse the *tna*-operon mathematical model and contrast its results with the experimental ones. Finally, some concluding remarks are brought forth in the last section.

## Tryptophanase Operon

**Figure 1:**
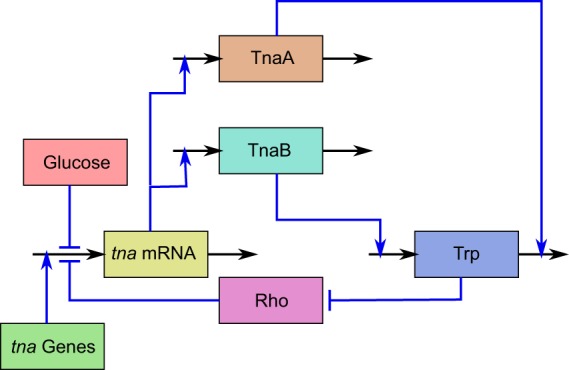
Schematic representation of the *tna* operon regulatory pathway in *Escherichia coli*. Rectangles represent chemical species, black incoming (outgoing) arrows represent production or intake (degradation or output), and blue lines represent regulatory interactions. Blue lines ending in arrows (hammer-heads) denote positive (negative) regulation. The regulatory pathway is as follows. *tna* mRNA is produced via transcription of the *tna* genes. *tna* mRNA is further translated to synthesize proteins TnaA and TnaB. Permease TnaB transports tryptophan (Trp) into the cell, while enzyme TnaA metabolises it. Furthermore, tryptophan inhibits protein Rho, which prematurely terminates transcription of the *tna* genes. Finally, glucose down-regulates transcription initiation via catabolite repression.

The *tna* operon of *E. coli* comprises two structural genes: *tnaA* and *tnaB*, that respectively code for the enzyme tryptophanase, which metabolises tryptophan (allowing it to be used as a carbon source), and for a tryptophan specific permease that uptakes this amino acid from the extracellular medium. The expression of these genes is positively regulated by extracellular tryptophan and negatively regulated by external glucose. Below we briefly review the mechanisms responsible for this regulation. We refer the reader to Fig. 1, where this operon regulatory pathway is schematically represented.

One of the regulatory mechanisms of the tryptophanase operon is catabolite repression. Without entering into the corresponding molecular details, this mechanism depends on the intracellular glucose level. When it is high, it indirectly leads to inhibition of adenylyl cyclase, and so cAMP levels are low. Contrarily, in the absence of glucose, the enzyme adenylyl cyclase is activated, and this in turn leads to high levels of cAMP. cAMP then binds to catabolite activator protein (CAP) and the resulting complex binds to a promoter sequence on the *tna* operon, enhancing transcription initiation^12^.

Another regulatory mechanism in the *tna* operon is termed Rho (*ρ*) mediated termination. This mechanism acts on the so-called *tnaC* leader sequence, which is a 24-bp long sequence located downstream of the *tna* promoter and upstream of genes *tnaA* and *tnaB*^13,14^. When protein *ρ* binds to a specific site on the *tnaC* mRNA, it uses ATP to power its helicase function and move along the transcript towards the polymerase (RNApol). Upon reaching the transcription complex, it forces the disengagement of mRNA from both DNA and RNApol. Rho mediated termination in the *tna* operon is suppressed when there is tryptophan available in the cytoplasm. This is due to a single Trp codon in the *tnaC* sequence. When the ribosome reaches this codon in the mRNA transcript, the arrival of a charged tRNA^Trp^ molecule triggers a complex structural change, which in turn leads to a stoppage of the translation complex, effectively blocking *ρ* advance^15,16^.

Gene *tnaB* codes for TnaB permease: a low affinity tryptophan porter protein. Mtr, along with AroP, are the only other known tryptophan permeases in *E. coli*, though the last one is not specific and also imports tyrosine and phenylalanine into the cytoplasm^17–19^. Tryptophan is a valuable and expensive amino acid, so it is expected to find several mechanisms to provide the cell with it, either by transportation or biosynthesis^18,20^; hence the different transporters. Despite this, TnaB has been proven to be the only main tryptophan transporter present under the conditions needed for expression of the *tna* operon, because the *mtr* gene is repressed by tryptophan itself^17,21^ and studies on *E. coli* with repressed or deleted *aroP* gene expression have proven no incidence over TnaB function^19,22^.

We can see from the discussion in the above paragraphs that a positive feedback loop is involved in the *tna*-operon regulatory pathway, which makes it a candidate to show bistability. As the expression of *tnaB* increases, more tryptophan is transported into the cytoplasm. This in turn decreases the probability of *ρ*-mediated termination, and increases the expression of the operon genes, thus closing the positive feedback loop.

Besides catabolite repression and *ρ*-mediated termination, there exists another regulatory mechanism at the post-translational level in the *tna* operon. Li and Young^23^ found that, just after been translated, protein TnaA localizes at the cell poles, where it forms a well delimited and tight focus. Although this kind of structures are usually assumed to be inclusion bodies, this possibility was ruled out in the case of TnaA foci via different tests involving isolation, purification and *in vivo* observation techniques^23^. Further studies revealed that during lag and exponential growth phases TnaA is inactive and clustered in foci. Once in the stationary phase, foci disperse and indole production begins, thus suggesting that tryptophanase activates at dispersion. In agreement with this, Li and Young^24^ proved that foci are mainly formed by inactive TnaA dimers and monomers, while the active form of this enzyme is a tetramer formed by four identical 52.8 kDa subunits. Further results suggest that TnaA foci disintegration is due to an unknown cAMP-CAP independent post-translational mechanism related to glucose^25^.

## Materials and Methods

**Figure 2:**
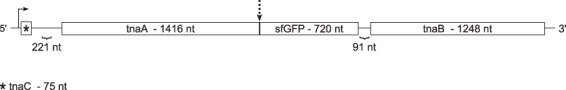
Schematic representation of the *tna*-operon DNA sequence in the *E. coli* GL69 strain, which includes a *tnaA-sfGFP* fusion. The folded arrow stands for the operon promoter at −24 nt from tnaC. The dotted arrow denotes the point in which the *sfGFP* gene sequence replaces the tnaA gene stop codon.

To study the dynamic behaviour of the *tna* operon in *E. coli*, we employed a strain of this bacterium (called GL69), which contains a *sfGFP* gene fused to gene *tnaA* within the bacterial genome, see Fig. 2. Thus, tryptophanase monomers in this bacterial strain are fused to super-folder Green Fluorescent Proteins, and cellular fluorescence levels might be used as a direct measurement of gene *tnaA* expression level. This last assertion depends on the assumption that the specific fluorescence of sfGFP is independent of whether the protein is fussed, dispersed or in foci, and independent of the foci size. There is not enough evidence to fully sustain this supposition, but we expect it to be valid due to the characteristics of sfGFP, which was designed to preserve its relative fluorescence regardless of the protein it is fused to. As a matter of fact, Pèdelacq *et al*.^26^ studied the behavior of sfGFP as a fusion tag, considering numerous different proteins. They found that sfGFP folds well, even when fused to poorly folded polypeptides, and consequently whole-cell fluorescence is in all cases directly proportional to the sfGFP-protein fusion molecule count.

Our experimental strategy consisted in growing *E. coli* GL69 in a micro-bioreactor under different medium conditions. We did this to drive bacterial cultures to a stationary state, both regarding bacterial density and *tna*-operon expression level. Then, we collected samples and analysed sfGFP fluorescence at the single cell level, via image analysis techniques. Furthermore, we developed a mathematical model for the *tna*-operon regulatory pathway, and analysed it to complement the experimental results.

Below we describe in detail the experimental materials and methods we employed, and derive the mathematical model.

### Strains and Growth Conditions

The *E. coli* GL69 strain^23^ was kindly provided by Kevin Young’s lab at University of Arkansas for Medical Sciences. Stocks were prepared in 15% glycerol and kept at −80 °C. Working stocks were kept in solid LB-Agar plates at 4 °C. Cultures were performed in M9 minimal medium, supplemented as stated by Li and Young^24^, with 18 mM sodium pyruvate instead of 1% Bacto-Casaminoacids. Culture media were further enriched with glucose and tryptophan as stated below. All media and stock solutions were filter sterilized.

Growth curves were performed for 15 h in 96-well culture microplates at 37 °C. OD600 was measured every 15 min. Tested glucose concentrations were {500, 250, 125, 62.5, 31.25, 15.62, 7.81, 3.9, 1.95, 0.97, 0.48, 0.24} mM, while tested tryptophan concentrations were {48.97, 24.48, 12.24, 6.12, 3.06, 1.53, 0.76, 0.38, 0.19, 0.09, 0.04, 0.02} mM. Maximum concentrations in both cases were chosen to ensure medium saturation. Each experiment was performed in triplicate.

### Micro-Bioreactor Fabrication

**Figure 3:**
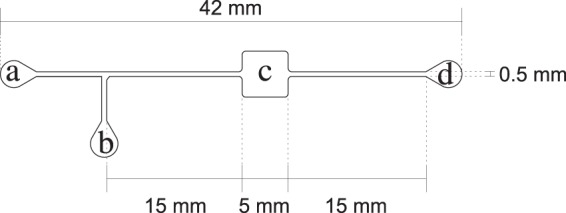
Upper view of the micro-reactor device designed to carry out continuous-flow *E. coli* cultures. This device consists of two inlets (**a**,**b**), a channel connecting both to a growth chamber (**c**) and an outlet (**d**), connected to the growth chamber by another channel. The channels and the growth chamber are 50 *μ*m high.

The bioreactor design was plotted in AutoCAD–AutoDesk^TM^–at a 1:1 scale (see Fig. 3). This design was later used to create a master mold to manufacture the devices through soft lithography.

A master mold was created on a Si wafer coated with SU-8 3050–Microchem^TM^, a negative photoresist. It was prepared as per manufacturer’s instructions to be 50 m high. The device design was etched via focused laser writing at 70 mW × 100%.

Micro-bioreactor devices were prepared by pouring degassed PDMS, mixed with curing agent in a 10:1 proportion, on the master mold and resting for polymerisation at 80 °C for 2 hours. After polymerization, devices were separated from the master mold and cored at the inlet and outlet locations with a 1.5 mm Uni-Core^TM^ tool. They were washed twice in MiliQ water for 30 minutes, then for 15 minutes in 97% isopropanol, and once again for 15 minutes in MiliQ water. All washes were performed in an ultrasonic bath sonicator. Finally, the devices were left to dry in a desiccator for 48 hours.

Dry bioreactors were assembled on microscope slides previously cleaned with Micro-90 cleaning solution–Cole-Parmer^TM^. Both PDMS molds and slide surfaces were treated with oxygen plasma generated by a BD-20AC Laboratory Corona Treater–Electro-Technic^TM^–for 30 to 60 seconds and, immediately after assembly, heated for up to 2 hours at 80 °C.

Assembled devices were perfused with 97% isopropanol, dried at 95 °C, perfused with 0.04% PVP-40 and dried again at 95 °C. After drying, devices were sterilized by autoclaving.

### Continuous-Flow Culture Setup

Bacteria were grown overnight (ON) at 37 °C in 5 ml of M9 minimal medium with sodium pyruvate, then harvested by centrifugation at 1 × g per 5 min, washed and centrifuged twice in 10 mM PBS. After the last centrifugation, supernatant was discarded and cells resuspended in 10 mM PBS. This cell suspension was later used to feed the micro-bioreactor.

All the media used to perform continuous flow experiments were based in the above described minimal M9 medium further supplemented with different concentrations of glucose–{0.0, 3.7, 7.5, 15.0, 30.0} mM, and tryptophan–{0.0, 6.0, 12.0, 24.0, 48.0} mM. To avoid the formation of cell clumps inside the micro-bioreactor, Tween20 was added to all media to a final concentration of 0.02%.

Sterile bioreactors were filled with medium using a kdScientific^TM^ KDS-210 syringe pump through outlet **c** at 150 l/min. Then, they were inoculated with 60 l of PBS-cells suspension at 120 l/min through inlet **b** (see Fig. 3), while occluding inlet **a** to avoid syringe contamination. After inoculation, medium was flowed through inlet **a** at 0.3 l/min. After 24 hrs, 5 l samples were taken from the outlets and loaded on 1% agarose coated slides for imaging.

### Microscopy

Bright field and fluorescence 10-second long videos from at least 10 different regions were obtained from each sample with a CCD Watec^TM^ WAT-902H2 Supreme camera mounted on an Olympus^TM^ BX51 microscope, with an 100 × oil objective (1.3 NA PH3), and illuminated by the Olympus^TM^ fluorescence source BH2-RFL-T3. sfGFP fluorescence was visualized through an Olympus^TM^ U-MNIB2 filter set (480/20 excitation/510 LP emission). Videos were recorded at 30 fps with a 720 × 480 pixels resolution.

Since we intend to use fluorescence as a measure of gene expression, we took special care of maintaining the same focus in all recorded frames, as a slight defocus can have a large effect on the measured expression level. Given that even a small change of focus changes the apparent size of bacteria, we measured (by means of image analysis techniques) the width of all bacteria in all of the recorded frames. Then, we computed the mean bacterial width in every frame, and contrasted these values among all frames. The resulting average value (averaged over all recorded frames) of mean bacterial widths is 1.02 m, while the corresponding standard deviation is 0.05 m. Thus, the observed regularity of bacterial widths among frames indicates that we kept a constant focus in all of our experiments.

### Image Analysis

200 frames of each video, both bright field and fluorescence, were averaged in order to enhance sharpness, reduce background noise, and acquire a representative single image from each recorded region. To quantify fluorescence intensity within each cell, images were analysed via a custom software developed in MatLab R2017b^TM^ (The MathWorks, Inc.). Below we briefly describe how this software works.

First, mask images were created from bacteria regions in bright field images. To determine what regions belonged to a cell body, a segregation operation was performed by analysing mean contrast and intensity. If contrast was good enough, a threshold was applied at half intensity via a simple binary operation (imbinarize in MatLab), in order to isolate the areas of interest. In the opposite case, the bright field image was added to the corresponding fluorescence image to improve contrast. Then, the binary segmentation function was performed with an adaptive sensitivity of 0.35. This process rendered images with empty areas corresponding to bacteria bodies, that is mask images.

The resulting masks were then subtracted from fluorescence images to only keep the pixels corresponding to bacteria. The average bacterium area was 190 pixels. To avoid considering debris and extraneous objects as bacteria, we applied lower (114 pixels) and upper (7600 pixels) area thresholds. These thresholds allow us to account for smaller than average cells, as well as clusters of up to 40 cells.

Once bacterium areas were isolated in the fluorescence images, the intensity of each pixel was quantified from the absolute difference between the masked and the original fluorescence images. To eliminate background fluorescence, the intensity of all pixels outside of bacteria was computed in the same way, and the average value was subtracted from all pixels corresponding to bacteria. Intensity values lower than 90 (in a scale from 0 to 255) were regarded as corresponding to spread fluorescence. Conversely, fluorescence values above 90 were considered as corresponding to foci fluorescence. From this data, we computed the spread and foci fluorescence densities in each bacterium by adding the intensities of the corresponding pixels, and dividing by the cell area.

It is important that the images are not over-saturated in order to have precise measurements of protein concentration. In this regard, the maximum value for the mean fluorescence intensity recorded in single foci, considering all recorded images, was 180. Since this is reasonably far from the saturation value: 255, we assure that our fluorescence measurements can be regarded as proportional to protein concentration.

### Model Development

In this subsection we introduce a deterministic mathematical model, based on ordinary differential equations, for the tryptophanase-operon regulatory pathway. There is plenty of evidence that mRNA half life is much shorter than that of proteins in prokaryotic organisms–see for instance^27^. This means that the *tna* mRNA dynamics are much faster than those of proteins TnaA and TnaB, and so it is safe to assume that mRNA levels reach equilibrium almost instantaneously as compared with slow protein changes. From this, a mass-action-based model for the *tna* operon regulatory pathway can be written as follows:

1 $$\dot{A}={k}_{A}{P}_{G}({G}_{e}){P}_{W}(W)-({\gamma }_{A}+\mu )A,$$

2 $$\dot{B}={k}_{B}{P}_{G}({G}_{e}){P}_{W}(W)-({\gamma }_{B}+\mu )B,$$

3 $$\dot{W}=(\alpha +\beta B){W}_{e}-(\delta +\epsilon A{P}_{A}({G}_{e},{W}_{e})+\mu )W.$$

In these equations, *A*, *B*, and *W* respectively denote the intracellular concentrations of tryptophanase enzyme (TnaA), of tryptophan permease TnaB, and of intracellular tryptophan. *P*_*G*_(*G*_*e*_) stands for transcriptional regulation via catabolite repression of the operon genes, *G*_*e*_ represents extracellular glucose concentration, function *P*_*W*_(*W*) accounts for tryptophan-mediated regulation via premature transcriptional termination, and *P*_*A*_(*G*_*e*_, *W*_*e*_) denotes the fraction of active tryptophanase enzymes in terms of external glucose and tryptophan levels. Parameters *k*_*A*_ and *k*_*B*_ represent the maximal production rates of proteins TnaA and TnaB, parameters *γ*_*A*_ and *γ*_*B*_ are the respective protein degradation rates, and parameter *μ* is the bacterial population growth rate. The term *αW*_*e*_ accounts for tryptophan uptake through permeases other than TnaB, while the term *βBW*_*e*_ stands for tryptophan uptake via TnaB. Finally, *δW* accounts for tryptophan consumption during protein synthesis, whereas $\epsilon$*AW* represents tryptophan catabolism by TnaA.

The model in Eqs (1–3) involves 9 parameters (*k*_*A*_, *k*_*B*_, *γ*_*A*_, *γ*_*B*_, *μ*, *α*, *β*, *δ*, $\epsilon$), plus those appearing in functions *P*_*G*_(*G*_*e*_), *P*_*W*_(*W*), and *P*_*A*_(*G*_*e*_, *W*_*e*_). Since there is not enough information to estimate all of these parameters from reported experimental data, it is convenient to find a way to reduce the number of effective parameters to facilitate the analysis of the system dynamic behaviour. This objective is achieved by simplifying and normalizing the model equations as follows. The mean half life of most bacterial proteins in *E. coli* is typically long in comparison with bacterial doubling time. This means that *γ*_*A*_, *γ*_*B*_ _≪_ *μ*, and so we can assume that *γ*_*A*_ + *μ*, *γ*_*B*_ + *μ* ≈ *μ*. From this, and defining the following dimensionless variables:

4 $$t^{\prime} =\mu t,\,a=A\mu /{k}_{A},\,b=B\mu /{k}_{B},\,w=\xi W,$$

(numerically, *ξ* equals *μ*/*α* but it has concentration units) the system of differential equations in Eqs (1–3) can be simplified as follows:

5 $$a^{\prime} ={\rho }_{G}({G}_{e}){\rho }_{W}(w)-a,$$

6 $$b^{\prime} ={\rho }_{G}({G}_{e}){\rho }_{W}(w)-b,$$

7 $$w^{\prime} =(1+\varphi b){W}_{e}-(\theta +\psi a{\rho }_{A}({G}_{e},{W}_{e}))w,$$

with *ρ*_*G*_(*G*_*e*_) = *P*_*G*_(*G*_*e*_), *ρ*_*W*_(*w*) = *P*_*W*_(*wα*/*μ*), *ρ*_*A*_(*G*_*e*_, *W*_*e*_) = *P*_*A*_(*G*_*e*_, *W*_*e*_), *ϕ* = *βμ*/*k*_*B*_*α*, *θ* = 1 + *δ*/*μ*, and *ψ* = $\epsilon$/*k*_*A*_. Notice that, as a result of the simplifying assumptions, Eqs (5) and (6) are identical. This means that variables *a* and *b* are redundant and so, the system in Eqs (5–7) can be reduced to the following differential equation system:

8 $$p^{\prime} ={\rho }_{G}({G}_{e}){\rho }_{W}(w)-p,$$

9 $$w^{\prime} =(1+\varphi p){W}_{e}-(\theta +\psi {\rho }_{A}({G}_{e},{W}_{e})p)w,$$

where *a* = *b* = *p*.

To complete the model we need expressions for functions *ρ*_*G*_(*G*_*e*_), *ρ*_*W*_(*w*), and *ρ*_*A*_(*G*_*e*_, *W*_*e*_). Regarding the first one, recall that extracellular glucose indirectly represses the expression of several genes involved in the metabolism of carbon sources. This is known as catabolite repression. To account for this, *ρ*_*G*_(*G*_*e*_) should be a monotonic decreasing function of *G*_*E*_ satisfying *ρ*_*G*_(0) = 1 and ${\mathrm{lim}}_{{G}_{e}\to \infty }{\rho }_{G}({G}_{e})=0$. To take this into account we assume that *ρ*_*G*_(*G*_*e*_) is a decreasing Hill function of the form^28^:

10 $${\rho }_{G}({G}_{e})=\frac{{K}_{G}^{{n}_{G}}}{{K}_{G}^{{n}_{G}}+{G}_{E}^{{n}_{G}}}\mathrm{.}$$

As for regulation of *tna*-operon expression via intracellular tryptophan, recall that intracellular tryptophan indirectly enhances the expression of the *tna*-operon genes by preventing premature Rho-factor-mediated transcriptional termination. From this, function *ρ*_*W*_(*w*) must be a monotonically increasing and satisfy *ρ*_*W*_(0) ≈ 0 and ${{\rm{l}}{\rm{i}}{\rm{m}}}_{w\to \infty }{\rho }_{W}(w)=1$. One way to comply with these requirements is to assume^28^:

11 $${\rho }_{W}(w)=\frac{{w}^{{n}_{W}}}{{K}_{W}^{{n}_{W}}+{w}^{{n}_{W}}}.$$

The only thing missing is an expression for function *ρ*_*A*_(*G*_*E*_, *W*_*E*_), but we do not have enough information to propose it at this moment.

Observe that after the simplification process, the number of effective parameters in Eqs (8) and (9) has been reduced from 9 (in the original model) down to 3 (*ϕ*, *ψ*, and *θ*), plus 4 parameters in the definitions of functions *ρ*_*G*_(*G*_*e*_) and *ρ*_*W*_(*w*), plus those that will appear in the definition of function *ρ*_*A*_(*G*_*e*_, *W*_*e*_).

## Experimental Results

As stated, the present work is aimed at studying the possible bistable behaviour of the *tna* operon of *Escherichia coli*, as well as improving our understanding of the recently discovered regulatory mechanism at the post-translational level. To achieve these objectives, we carried out continuous-flow bacterial cultures, using the micro-bioreactor schematically represented in Fig. 3, for long enough periods of time to ensure a true stationary state (see section B of the appendix for an experimental proof of this assertion).

In our experiments we employed *E. coli* GL69, which was derived from *E. coli* MG1655 by fusing gene *sfGFP* to gene *tnaA*. Hence, as discussed in the Materials and Methods section, fluorescence intensity can be used as a measurement of the expression level of gene *tnaA* at the single-cell level. We investigated with this strain the expression of gene *tnaA* under various stationary levels of tryptophan and glucose in the culture medium. However, before doing this, it was necessary to set the ranges of tryptophan and glucose concentrations to be considered. See Section A of the Supplementary Information for a detailed description of the experiments we performed to achieve this objective.

### Stationary *tna* Operon Dynamics

After validating our experimental protocol, we investigated the steady-state behaviour of *E. coli*’s *tna* operon under different levels of environmental glucose and tryptophan. To do this, we followed the methodology described in the Materials and Methods section to culture *E. coli* GL69 under continuous flow conditions. Those cultures were perfused with M9 medium supplemented with 18 mM pyruvate, plus combinations of tryptophan (*W*_*e*_) and glucose (*G*_*e*_) concentrations in the following sets: *W*_*e*_ ∈ {0, 6, 12, 24, 48} mM and *G*_*e*_ ∈ {0, 3.75, 7.5, 15, 30} mM.

All cultures were left to evolve for 24 hrs after inoculation so the system reached a steady state (Supplementary Fig. 3), and then a sample was collected from the micro bioreactor. From each environmental condition we photographed a little more than 1000 bacteria (about 350 per independent experiment) under the fluorescence microscope, and then we analysed bacterial fluorescence at the single-cell level by means of an image-analysis custom software that we implemented in Matlab–see the Materials and Methods section.

**Figure 4:**
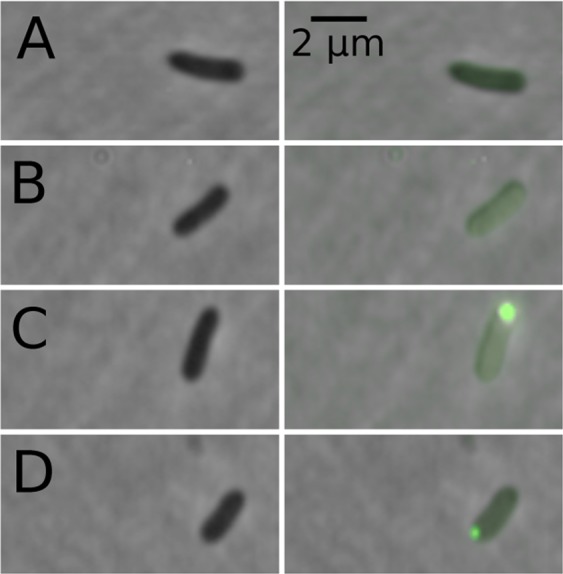
Photographs of cells sampled from the continuous flow cultures here reported. Images in the left column were taken with a phase contrast objective, while the images on the right column represent to the same bacteria as those in the left, but in them the corresponding fluorescence (with background fluorescence subtracted) and the phase contrast images have been overlaid. These images illustrate the different types of observed cells (**A**) almost zero fluorescence, (**B**) distributed fluorescence, (**C**) distributed fluorescence plus fluorescence concentrated in a focus, (**D**) almost all fluorescence concentrates in a focus.

In Fig. 4 we show representative photographs of *E. coli* GL69 cells in different states of *tna*-operon expression. Notice how in some cases bacteria present very little to no fluorescence. In some other cases, cell fluorescence is concentrated in a single focus located at the pole. It is also possible that cell fluorescence is homogeneously dispersed across the cell body; or even that a fraction of such fluorescence is concentrated in a focus while the rest is dispersed.

We performed two different measurements in every single cell: (a) fluorescence density, which we estimated by normalizing the total fluorescence within each cell by the cell area, and (b) fraction of dispersed fluorescence, computed as the ratio of dispersed to total (dispersed plus foci) fluorescence.

**Figure 5:**
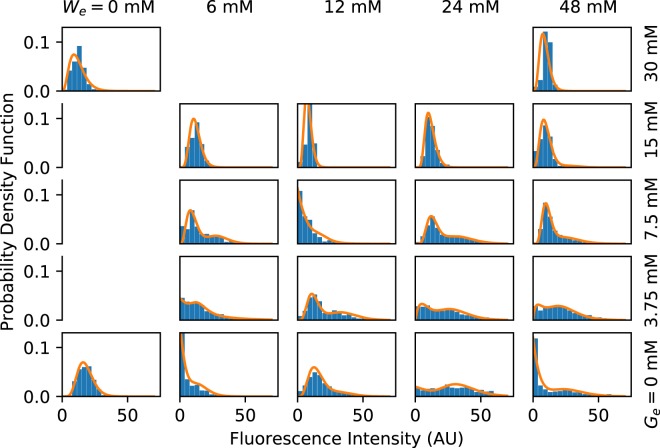
Probability density functions (PDFs) computed from single-cell fluorescence measurements (bar plots), carried out in samples from continuous-flow cultures growing in media with different concentrations of tryptophan (*W*_*e*_) and glucose (*G*_*e*_). The experimental results were fitted to mixture distributions of the form *ω*Γ(*x*; *α*_1_, *β*_1_) + (1 − *ω*)Γ(*x*; *α*_2_, *β*_2_), where Γ(*x*; *α*, *β*) is a gamma PDF with parameters *α* and *β*. Fitting curves are represented in all cases with solid lines.

First, we analysed the total sfGFP fluorescence density at the single cell level. As discussed in the Materials and Methods section, this measurement can be regarded as proportional to the expression level of the *tna* operon, regardless of the TnaA enzymatic activity. In Fig. 5 we show the probability density functions (PDFs) for the single-cell sfGFP fluorescence corresponding to every one of the experimentally tested combinations of tryptophan and glucose levels in the culture medium. The results of the three independent experiments performed for each combination of environmental glucose and tryptophan were grouped, after verifying repeatability, to compute these PDFs. A few interesting facts about the *tna* operon are apparent: (A) As expected, as the concentration of tryptophan in the medium increases, so does the probability of observing cells with higher fluorescence (*tna* expression) levels. However, higher tryptophan levels are necessary to observe a similar effect when the medium glucose concentration is augmented. Interestingly, when glucose concentration in the medium is very high (about 30 mM), the *tna* operon is never induced, even with the highest tryptophan concentration employed (48 mM). (B) Several of the PDFs are bimodal. This can be better appreciated by the fact that bimodal distributions can be fitted by mixture distributions of the form *ω*Γ(*x*; *α*_1_, *β*_1_) + (1 − *ω*)Γ(*x*; *α*_2_, *β*_2_), where Γ(*x*; *α*, *β*) is a gamma PDF with parameters *α* and *β*. These bimodal PDFs suggest that the *tna* operon shows a bistable behaviour for some ranges of tryptophan and glucose medium concentrations. Bimodal PDFs are observed in the range [0, 48] mM of tryptophan, for glucose concentrations smaller than or equal to 7.5 mM. When glucose concentration equals 15 mM, a bimodal distribution is observed only at 48 mM tryptophan concentration, and no bimodal distribution is observed at 30 mM glucose. (C) The *tna* operon is never completely shutoff. Instead, there is a basal expression level, even when there is no tryptophan in the medium and glucose concentration is high. This indicates that, even when catabolite repression is fully active, the probability that the *tna* promoter is non-negligible; and that Rho mediated termination never achieves a 100% efficiency. Since the *tna* operon is subject to positive feedback regulation, this basal expression could be a mechanism to speed operon activation.

**Figure 6:**
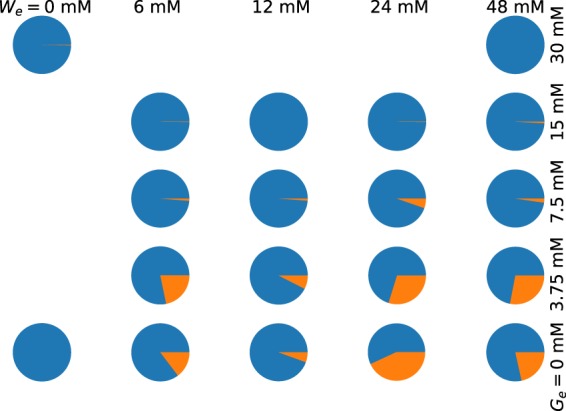
Pie plots representing the mean fractions of dispersed fluorescence (blue) and of florescence in foci (orange), averaged over all sampled bacteria growing under continuous-flow conditions in media with different concentrations of tryptophan (*W*_*e*_) and glucose (*G*_*e*_).

Regarding the fraction of dispersed fluorescence in single cells, we also verified the repeatability of our experimental setup and lumped together the data obtained from the three experiments of every medium condition. Then, we computed the average fraction of dispersed fluorescence (and also the average fraction of fluorescence in foci). The obtained results are shown in Fig. 6. Notice that the extracellular levels of both tryptophan and glucose affect the stability of TnaA foci. Li and Young^25^ reported that the addition of glucose favours the disappearance of TnaA foci. However, to the best of our knowledge, this is the first report of a similar effect due to tryptophan. The fact that we explored numerous tryptophan and glucose concentrations allowed us to recognize that foci-stability regulation by these carbon sources is more complex than originally reported by Li and Young. Our results suggest that moderate increments on the level of either tryptophan or glucose in the culture medium favours foci formation, but further increments have the opposite effect.

**Figure 7:**
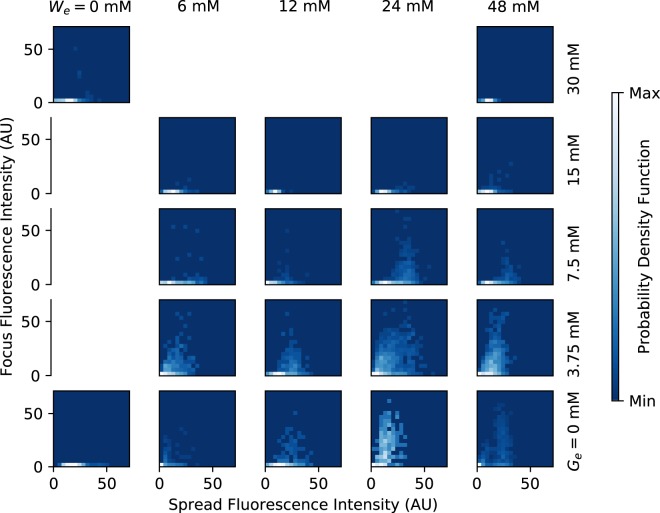
Plots of the probability density functions that a single bacterium has given values of dispersed and foci fluorescence in media with different concentrations of tryptophan (*W*_*e*_) and glucose (*G*_*e*_).

A more detailed analysis of the dependence of foci formation on the medium conditions is presented in Fig. 7, where the probability density function that a bacterium has given values of foci and dispersed fluorescence is plotted for different tryptophan and glucose concentrations. There, we can see foci form only when the expression level of the *tna* genes is high. In other words, when there is a small amount of TnaA enzymes in the cytoplasm, all of them are active; but when there are many of them, they can be either active or inactive with a probability distribution that depends on the medium conditions.

## Mathematical Modelling Results

As discussed in the former section, our experimental results suggest that the *tna* operon of *E. coli* displays bistable behaviour for some concentrations of tryptophan and glucose in the culture medium, and that the enzymatic activity of TnaA is regulated in a complex way by both carbon sources. To test the compatibility of these results with the known regulatory pathway of this operon, and to further improve our understanding of its regulation, we analyse the mathematical model introduced in the Materials and Methods section, in which a definition for function *ρ*_*A*_(*G*_*e*_, *W*_*e*_) is missing.

To complete the mathematical model, let us take into account that, according to our experimental results, formation and dissociation of TnaA foci in bacterial cytoplasm is regulated in a complex way by both external tryptophan and glucose. In the absence of glucose, when external tryptophan is zero (*W*_*e*_ = 0), all TnaA proteins seem to be disperse. Small increases of *W*_*e*_, apparently promote foci formation. However, all foci dissociate at even higher *W*_*e*_ values–see Fig. 6. A similar behaviour is observed when external glucose concentration changes, although the concentrations at which foci form and dissociate differ from those of external tryptophan. If we consider that, according to Li and Young^24^, TnaA is inactive (active) when it is located in foci (dispersed), we can estimate from our results the fraction of active TnaA (disperse fluorescence) at different concentrations of external tryptophan and glucose. After considering numerous possible functions, we were able to reproduce most of the features of the experimental data with the following expression for *ρ*_*A*_(*G*_*e*_, *W*_*e*_):

12 $${\rho }_{A}({G}_{e},{W}_{e})=1-{(x/{K}_{1})}^{3}\,\exp (\,-\,x/{K}_{2}),$$

13 $$x=\lambda {G}_{e}+{W}_{e}.$$

The right hand side of Eq. (12) converges to 1 at *x* = 0 and *x* → ∞, and has a single minimum in between. The value and position of this minimum are determined by *K*_1_ and *K*_2_. This behaviour agrees with the experimental evidence about foci formation and dissociation as the concentration of either external glucose or external tryptophan is increased. The way *G*_*e*_ and *W*_*e*_ are combined in Eq. (13) is consistent with the assumption that external glucose and/or tryptophan concentrations are transduced into an intracellular variable (i.e. a second messenger), which in turn regulates foci formation and dissociation.

### Steady State Analysis

All the measurements reported in the present paper were performed while the system is in the steady state. Thus, in order to have equivalent simulation results, we need to analyse the fixed points of the system given by Eqs (8–13). By definition, a steady state is a solution of the model equations that satisfies *a*′ = *p*′ = 0. It follows from this and Eqs (8) and (9) that

14 $${p}^{\ast }={\rho }_{G}({G}_{e}){\rho }_{W}({w}^{\ast }),$$

15 $$(1+\varphi {p}^{\ast }){W}_{e}=(\theta +\psi {p}^{\ast }{\rho }_{A}({G}_{e},{W}_{e})){w}^{\ast },$$

where w^*^ and p^*^ denote steady state values of the corresponding variables. Further substitution of Eq. (14) into Eq. (15) leads to the following equation for w^*^:

16 $$(1+\varphi {\rho }_{G}({G}_{e}){\rho }_{W}({w}^{\ast })){W}_{e}=(\theta +\psi {\rho }_{G}({G}_{e}){\rho }_{W}({w}^{\ast }){\rho }_{A}({G}_{e},{W}_{e})){w}^{\ast }.$$

The left hand side of Eq. (16) is a sigmoid monotonically-increasing function of w^*^ that asymptotically approaches to (1 + *ϕρ*_*G*_(*G*_*e*_))*W*_*e*_ as w^*^ tends to infinity. On the other hand, the right hand side is a monotonically increasing function of w^*^ that linearly diverges as (*θ* + *ψρ*_*G*_(*G*_*e*_))w^*^ when ${w}^{\ast }\gg {K}_{W}$. It follows from this that plots of the left and right hand sides of the last equation could intersect at up to three points, thus giving rise to bistability. In the following subsection we explore the system bistable behaviour in more detail.

### Parameter Estimation and Comparison with Experimental Data

Given that the *tna* operon has not been extensively studied as, for instance, the *trp* and *lac* operons, or the phage *λ* switch, there is not enough experimental data to estimate all of the model parameters. Furthermore, all of the reduced parameters of the normalized model are in terms of at least one parameter that cannot be estimated from experimental data. From these considerations, we decided to estimate the model parameters by fitting to our own experimental data.

**Figure 8:**
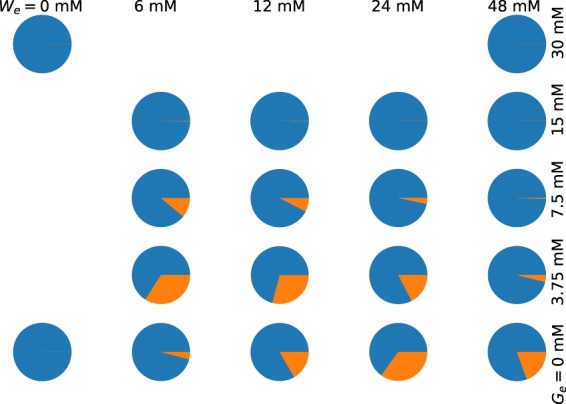
Pie plots illustrating the fractions of active (blue) and inactive (orange) TnaA enzymes, under different combinations of external tryptophan and glucose concentrations, as predicted by our mathematical model.

First, we started with parameters *K*_1_, *K*_2_, and *ξ* in function *ρ*_*A*_(*G*_*e*_, *W*_*e*_)–Eqs (12) and (13). By trial and error we found that setting:

17 $${K}_{1}=14,\,{K}_{2}=9,\,{\rm{and}}\,\xi =7,$$

makes the model qualitatively reproduce our experimental results. In Fig. 8 we show the model predictions for the mean fraction of active (dispersed) TnaA in different conditions of external tryptophan and glucose. By comparing with Fig. 5 we can appreciate that the mathematical model is able to qualitatively reproduce several of the corresponding experimental results. Namely: (A) When *G*_*e*_ = 0, the fraction of active enzymes decreases from *W*_*e*_ = 0 mM up to *W*_*e*_ = 24 mM, and then increases from *W*_*e*_ = 24 mM to *W*_*e*_ = 48 mM. (B) When *W*_*e*_ = 6, 12 mM, the fraction of active enzymes decreases from *G*_*e*_ = 0 mM to *G*_*e*_ = 3.75 mM, and then increases from *G*_*e*_ = 3.75 mM to *G*_*e*_ = 7.5 mM and beyond. The vast majority of TnaA enzymes are active when *G*_*e*_ = 15 mM. (C) When *W*_*e*_ = 24 mM, the fraction of active enzymes monotonically increases together with *G*_*e*_. The vast majority of TnaA enzymes are active when *G*_*e*_ = 15 mM.

After contrasting the model behaviour with the experimental results fraction of active TnaA enzymes, we analysed the model steady states. We programmed a custom algorithm in Python that makes use of the secant method^29^ to compute the roots of Eq. (16) for a given set of *W*_*e*_ and *G*_*e*_ values–recall that each one of these roots corresponds to a steady state, and we used this algorithm to compute the system steady states at many different (*W*_*e*_, *G*_*e*_) values. With the results we constructed a bifurcation diagram, in which the regions in the (*W*_*e*_, *G*_*e*_) parameter space where the system is monostable or bistable are clearly delimited. By trial and error we looked for a set of values for parameters *ϕ*, *ψ*, *θ*, *K*_*W*_, *n*_*W*_, *K*_*G*_, *N*_*G*_ such that we could reproduce the experimentally observed bistability region. The parameter values obtained in such a way are:

18 $$\varphi =150,\,\psi =0.1,\,\theta =6,\,{K}_{W}=60\,{\rm{mM}},\,{n}_{W}=4,\,{K}_{G}=11,\,{n}_{G}=4.$$

**Figure 9:**
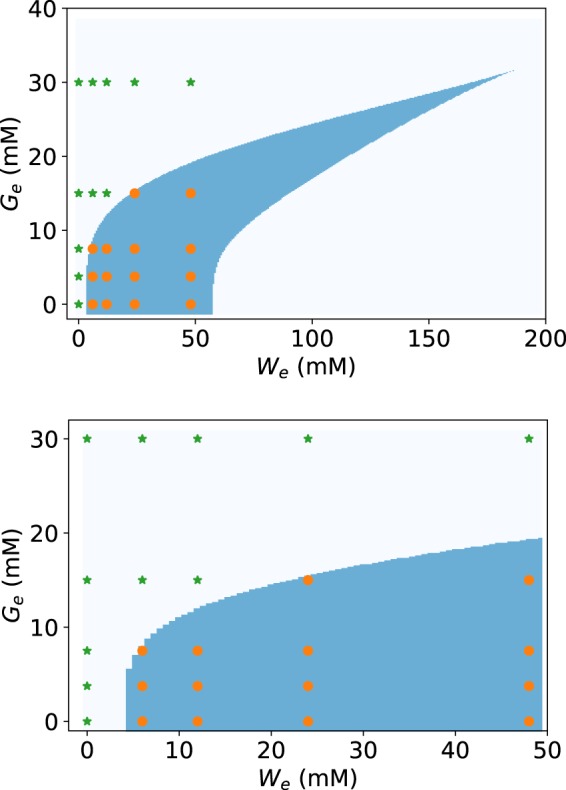
Bifurcation diagram in the *G*_*e*_ vs. *W*_*e*_ parameter space for the system of ordinary differential equations given by Eqs (8) and (13). In the bottom graph, the area corresponding to the experimental *W*_*e*_ and *G*_*e*_ ranges is zoomed. The blue-shaded region on both graphs encloses the *G*_*e*_ and *W*_*e*_ parameter values that give rise to bistability. The region to the left (right) of blue-shaded corresponds to the monostable uninduced (induced) *tna* operon steady-state. Finally, the (*W*_*e*_, *G*_*e*_) values at which our experiments were performed are denoted with green stars (if the corresponding experimental gene-expression PDFs are unimodal) and orange circles (if the corresponding PDFs of gene expression are bimodal).

In Fig. 9 we show the bifurcation diagram obtained with these parameter values. Note the good agreement between the model (Fig. 9 bottom graph) and the experimental (Fig. 4) results concerning the (*W*_*e*_, *G*_*e*_) values at which the system shows either a monostable-uninduced or bistable behaviour.

To further investigate the robustness of the bistable behaviour predicted by the mathematical model, we carried out a parameter sensitivity analysis as follows. First we fixed the values of *n*_*W*_, *n*_*G*_ = 4 because these parameters determine the maximum steepness of Hill-type regulatory functions^28^, and a value of 4 is enough for bistability to arise. Then, we randomly generated values for the resting 5 model parameters (*ϕ*, *ψ*, *θ*, *K*_*W*_, *K*_*W*_) from uniform probability distributions in the range from zero to twice the values reported in Eq. 18. Finally, we tested whether, with these randomly generated parameter sets, the model predicted a bistability region in the (*W*_*e*_, *G*_*e*_) space that matched our experimental results. That is, the (*W*_*e*_, *G*_*e*_) points for which we obtained bimodal gene-expression PDFs lie within the bistability region, while (*W*_*e*_, *G*_*e*_) points with unimodal probability density functions (PDFs) lie outside. In total, we found that ≈2,000 out of the 100,000 randomly generated parameter sets predicted bistability regions that agreed with the experimental results. From these parameter sets we computed histograms for each one of the parameters and show the results in the appendix, in Fig. 4.

The results in Fig. 4 of the Supplementary Information suggest the existence of bounded intervals for some parameter values (*θ*, *K*_*W*_, *K*_*G*_) such that the modelling and experimental results regarding bistability agree, while no upper bound is apparent for parameters *ϕ* and *ψ*. In either case, these results demonstrate that there is at least one none negligible region in the parameter space such that any parameter set in this region allows the model to reproduce the experimentally observed bistable behaviour.

In the Supplementary Information, in Fig. 5, we show scatter plots of the ≈2,000 randomly-generated parameter sets {*ϕ*, *ψ*, *θ*, *K*_*W*_, *K*_*G*_} with which the model predicts bistability regions in the *G*_*e*_ vs. *W*_*e*_ parameter-space that agree with experimental data. These scatter plots are projected onto the 10 possible 2-dimensional subspaces of the parameter space to illustrate potential correlations between different parameters. With the exception of parameters *θ* and *K*_*G*_, which are clearly negatively correlated, little correlation is observed between all parameter couples.

In summary, we have developed a mathematical model that is able to reproduce our experimental results regarding *tna*-operon bistability, and regulation of tryptophanase activity by extracellular glucose (*G*_*e*_) and tryptophan (*W*_*e*_). This reinforces our claim that the *tna* operon of *E. coli* displays bistability for a well defined region of the (*W*_*e*_, *G*_*e*_) parameter space. Furthermore, our model suggests that TnaA activity is probably regulated by extracellular tryptophan and glucose via a signalling pathway, which is activated by either glucose or tryptophan, albeit with different efficiencies.

## Concluding Remarks

We have studied the dynamic behaviour of *E. coli*’s *tna* operon. We did this both experimentally and with a mathematical model for this operon regulatory pathway. In particular, we were interested in assessing bistability, as well as improving our understanding of the post-translational regulation of TnaA activity.

Our experimental results suggest that the *tna* operon of *E. coli* presents a bistable behaviour in a well-defined region of the (*W*_*e*_, *G*_*e*_) parameter space. This claim is supported by the fact that we were able to reproduce this finding with a mathematical model that considers all the known mechanisms of the *tna*-operon regulatory pathway.

As discussed in the Introduction, the fact that (unlike the lactose operon) the tryptophanase operon is bistable, adds to the discussion of whether bistability is an optimal solution to the problem of rapidly and efficiently switching carbon sources. In this regard, observe in Figs 5 and 9 that the *tna* operon state enters the bistable region as the concentration of external tryptophan increases, but does not switch to the monostable induced state, even with tryptophan concentrations as high as 48 mM (take into consideration that this is an extremely high concentration; indeed we chose it because it corresponds to the largest amount of tryptophan that can be dissolved in M9 medium at room temperature). This disagrees with the switch-like behaviour, associated with bistability as a mechanism for switching carbon sources, which demands that the system flips from the monostable uninduced state to the monostable induced state as the inducer concentration increases. Then, if bistability is not the mechanism behind a switch-like behaviour in this case, the question of what dynamical role it may be playing naturally arises. Much work is needed to answer this question, but we believe it may be related to the bacterial-population fitness. As it has been discussed elsewhere^30^, heterogeneity could be an adaptive trait of microbial populations that inhabit constantly changing environments. In our case, due to bistability there is always a subpopulation of bacteria in which the *tna* operon is uninduced, even when tryptophan level is high and there is no glucose in the environment. This might secure a rapid adaptation of the bacterial population to nutritional shifts that involve a depletion of environmental tryptophan.

About the post-translational regulation of TnaA activity, our experimental results indicate that it is controlled by extracellular tryptophan and glucose. Li and Young^24^ had already demonstrated that glucose participates in this regulation. To our knowledge, this is the first report that shows that tryptophan also plays a regulatory role. Furthermore, our modelling results are consistent with the assumption that extracellular glucose and tryptophan regulate TnaA activity via a common signalling pathway, although they activate it with different efficiencies. Regarding the dynamical role played by the regulation of TnaA activity, it may be that it is similar to that of the enzyme inhibition regulatory mechanism in the tryptophan operon. It has been suggested in the last case^31^ that enzyme inhibition helps bacteria to adapt to environmental nutritional changes that occur more rapidly than the response time of gene expression. Although, further work is needed to prove or disprove this hypothesis, we wish to point out that it is consistent with the results in Fig. 7, which suggest that foci only form when there is a high expression of the *tna*-operon genes. In other words, when there is a small amount of TnaA enzymes, the fact that most of them are active favours a rapid operon activation due to the positive feedback regulation. On the other hand, if there is a large amount of enzymes, foci formation may be an efficient and rapid mechanism to regulate their activity as medium conditions change.

## Supplementary information

Supplementary information
