# Supplementary material for: Bistable behaviour and medium-dependent post-translational regulation of the tryptophanase operon regulatory pathway in Echerichia coli
Source: Sci Rep. 2019 Apr 1;9:5451. doi: 10.1038/s41598-019-41856-0 (PMC6443796; doi:10.1038/s41598-019-41856-0)
Supplement: Supplementary file 1 — Supplementary information [file 41598_2019_41856_MOESM1_ESM.pdf]

Bistable behaviour and medium-dependent post-translational regulation of the tryptophanase operon regulatory pathway in *Echerichia coli*

David I. Orozco-Gómez[1] Juan Eduardo Sosa-Hernández[2]

Óscar Adrián Gallardo-Navarro[1] Jesús Santana-Solano[1] Moisés Santillán[1*]

1. Centro de Investigación y de Estudios Avanzados del IPN, Unidad Monterrey, Parque PIIT, 66600 Apodaca NL, MÉXICO.
2. Tecnologico de Monterrey, School of Engineering and Sciences, Campus Monterrey, Ave. Eugenio Garza Sada 2501, 64849 Monterrey NL, MÉXICO.

A Establishment of culture-medium conditions

Since we wanted to consider the widest possible ranges of tryptophan and glucose concentrations, we looked for the maximum concentrations of these reagents that can be dissolved in water at room temperature without compromising bacterial growth. To do this we carried out in triplicate bacterial batch cultures in M9 medium supplemented with different concentrations of glucose or tryptophan, and measured the optical density (OD_{600}) at different times after inoculation. Take into account that OD_{600} can be regarded as proportional to the volumetric density of bacterial cells in the culture medium. All of the obtained OD_{600} vs. time curves presented the characteristic sigmoid shape, and thus we characterised them by means of two parameters: the culture doubling time during the exponential growth phase, T (which we computed by fitting the data corresponding to the first two culture hours to an exponential function), and the maximum optical density reached during the 15 hours the culture lasted (K). The obtained results for M9 medium supplemented with different amounts of glucose or tryptophan are presented in Fig. 1.

Observe in Fig. 1A that parameter K rapidly increases with glucose concentration until it reaches a maximum at about 30 mM, and then slowly decreases. Moreover, Fig. 1B shows that the culture doubling time is more or less constant for the whole range of glucose concentrations. From these results we decided to use the following glucose concentrations in our continuous-culture experiments: $G_e = \{0, 3.75, 7.5, 15, 30\}$ mM.

We can appreciate in Fig. 1C-D that parameter K increases while the doubling time decreases as the tryptophan concentration increases, until both of them stabilise at about 10 mM. Thus, we decided to employ the following tryptophan concentrations in the continuous-flow experiments: $W_e = \{0, 6, 12, 24, 48\}$ mM.

As discussed, one of the culture media to be considered in our experimental protocol is M9 medium supplemented with neither glucose nor tryptophan. Bacterial growth in such conditions is unlikely. Thus, we needed to incorporate another energy and carbon source that does not induce

*Correspondence to: msantillan@cinvestav.mx

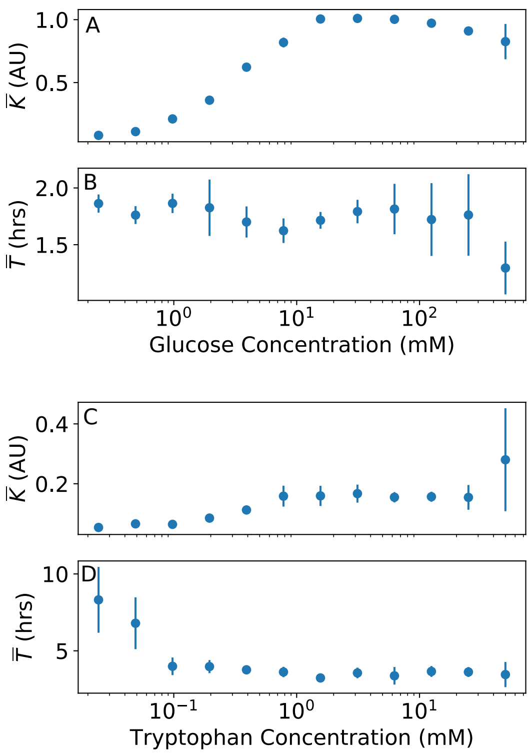

Figure 1: Mean doubling time (T) and maximum OD_{600} (K) for bacterial cultures growing in M9 minimal medium supplemented with different glucose concentrations (A, and B), and in M9 minimal medium supplemented with different tryptophan concentrations (C and D). Error bars denote standard deviation.

catabolite repression, and we chose pyruvate. To test whether pyruvate affects bacterial growth, we performed batch cultures with the following media: M9 + 18 mM pyruvate (P), M9 + 18 mM pyruvate + 48 mM tryptophan (PW), and M9 + 18 mM pyruvate + 30 mM glucose (PG). Three independent experiments were carried out per medium, and the culture optical density was measured at different times after inoculation in each experiment. We then computed the duplication time (T) and the maximum OD_{600} during the 15 hours the culture lasted, and averaged the results for each medium condition. The results are shown in Fig. 2. There, we can appreciate that while the medium supplemented with pyruvate allows bacteria to grow, this reagent does not affect noticeably cell growth in the media supplemented with tryptophan and glucose (compare with Fig. 1). Based on these results, all culture media in the forthcoming experiments were supplemented with 18 mM pyruvate, even when it is not explicitly stated.

Further analysis of Figs. 1 and 2 reveals that the doubling time in M9 medium supplemented with glucose is never lower than 1 hr, while in M9 medium supplemented with tryptophan is never lower than 4 hrs. These quite long doubling times can be explined by the fact that M9 is a poor medium, even when supplemented with glucose or tryptophan. To corroborate this assertion, we cultured the GL69 strain in LB medium and measured a doubling time of about 42 min, which agrees with the experiments reported by Li and Young [1]

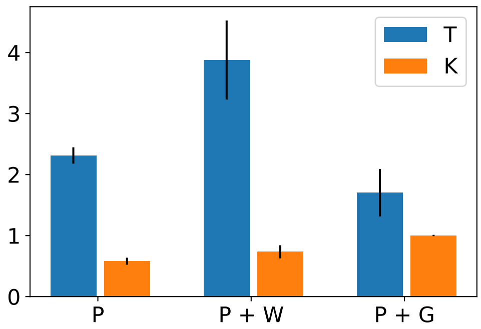

Figure 2: Mean doubling time (T) and maximum OD_{600} (K) for bacterial cultures growing in M9 minimal media supplemented with: pyruvate (P), pyruvate and tryptophan (P + W), and pyruvate and glucose (P + G). Error bars denote standard deviations.

B Validation of continuous-flow culture protocol

Before employing the micro-bioreactor represented in Fig. 3 of the main text it was necessary to test whether it allows bacterial cultures to achieve a stationary state. We implemented continuous-flow cultures in two different media: M9 + 18 mM pyruvate + 30 mM glucose, and m9 + 18 mM pyruvate + 48 mM tryptophan, and took samples at different times after inoculation to characterise the culture state. Three independent experiments were performed for each medium. From each sample we measured the optical density (OD_{600}) and, as explained in the Materials and Methods section, the sfGFP fluorescence density in several individual cells. To characterize the expression level of the *tna* operon in each experiment, we averaged the fluorescence density in all sampled cells. The resulting mean OD_{600} and mean average fluorescence density, averaged over the three repetitions for each medium, are plotted vs. time in Fig. 3.

Notice in Fig. 3A-B that the bacterial population reaches a stationary state at about 20 hrs after inoculation. Similarly observe in Fig. 3C-D that the average fluorescence intensity achieves a steady state in less than 40 hrs. after inoculation. These results corroborate that, as expected, the population-size and the *tna*-operon expression-level of bacteria cultured in the micro bioreactor reach steady values; and that 24 hrs. is a long enough time interval for that purpose. Henceforth, in all the forthcoming experiments, we waited for 24 hrs. after inoculating the micro-bioreactor before taking culture samples to perform measurements.

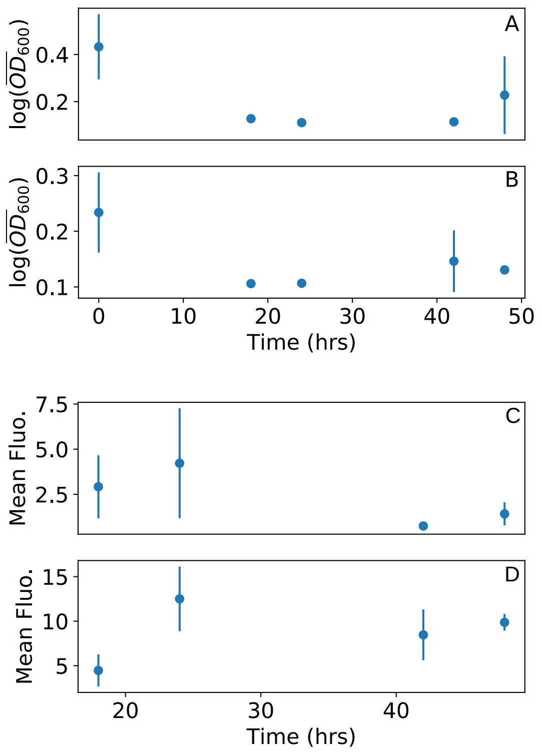

Figure 3: Mean optical density at 600 nm of bacterial cultures growing in the micro-reactor depicted in Fig. 3 of the main text at different times after being inoculated. Error bars denote standard deviation. A) M9 minimal medium + Glucose (30 mM). B) Minimal medium + Tryptophan (48 mM). Mean fluorescence intensity (averaged from three different experiments) of bacterial cultures growing in the micro-reactor depicted in Fig. 3 of the main text at different times after being inoculated. Error bars denote standard deviation. C) M9 minimal medium + Glucose (30 mM). D) Minimal medium + Tryptophan (48 mM).

C Robustness of bistability to parameter variations

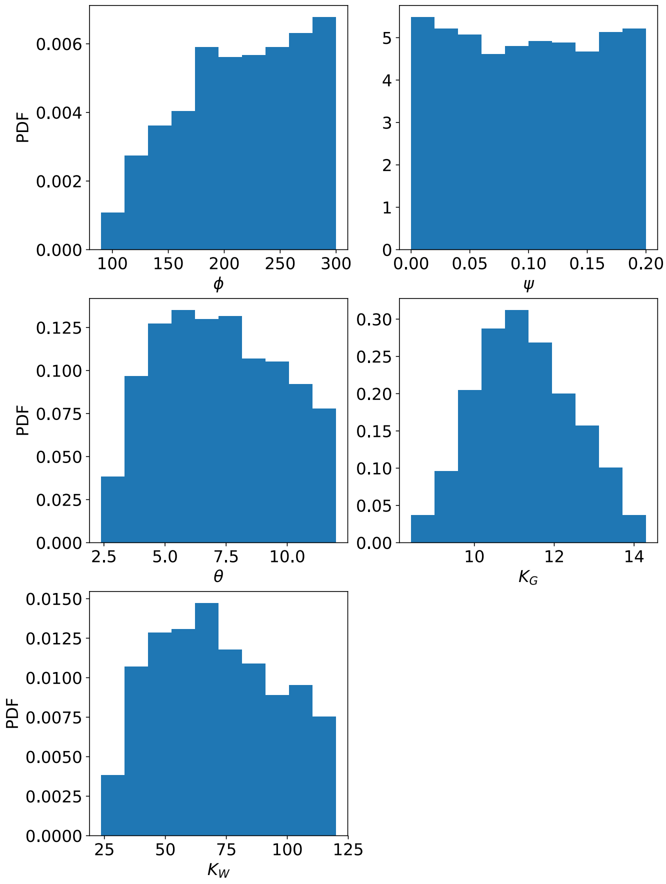

Figure 4: Probability density functions computed for the values of parameters $\pi, \psi, \theta, K_W, K_G$, obtained from about 2,000 randomly-generated parameter sets with which the model predicts bistability regions in the (W_e, G_e) space that are compatible with our experimental results.

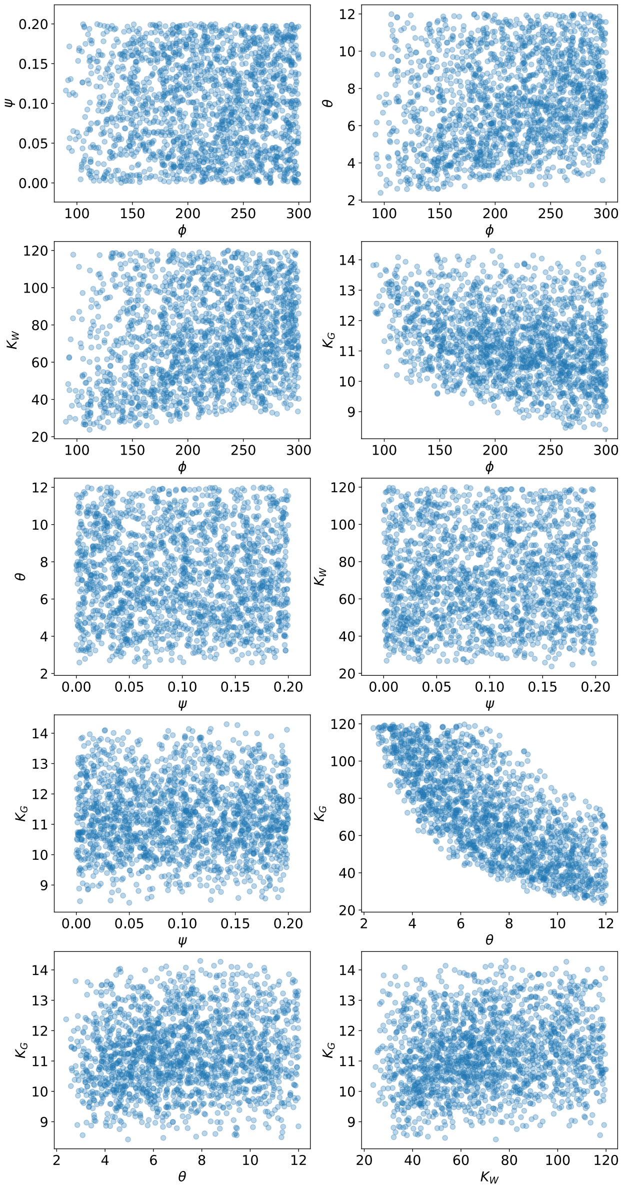

Figure 5: Scatter plots (proyected onto the 10 possible 2-dimensional subspaces of the parameter space) of 2,000 randomly-generated parameter sets $\{\phi, \psi, \theta, K_W, K_G\}$ that predict bistability regions in the G_e vs. W_e parameter space that agree with experimental data.

References

[1] Li, G. & Young, K. D. A new suite of tnaA mutants suggests that escherichia coli tryptophanase is regulated by intracellular sequestration and by occlusion of its active site. *BMC Microbiology* **15**, 14 (2015). URL `https://doi.org/10.1186/s12866-015-0346-3`.
